# Chronic Graft Loss and Death in Patients With Post-Transplant Malignancy in Living Kidney Transplantation: A Competing Risk Analysis

**DOI:** 10.5812/numonthly.14302

**Published:** 2014-03-10

**Authors:** Mahmoud Salesi, Zohreh Rostami, Abbas Rahimi Foroushani, Ali Reza Mehrazmay, Jamile Mohammadi, Behzad Einollahi, Saeed Asgharian, Mohammad Reza Eshraghian

**Affiliations:** 1 Department of Epidemiology and Biostatistics, School of Public Health, Tehran University of Medical Sciences, Tehran, IR Iran; 2 Nephrology and Urology Research Center, Baqiyatallah University of Medical Sciences, Tehran, IR Iran; 3 Department of Psychology, Faculty of Humanities, Tarbiat Modares University, Tehran, IR Iran; 4 Salamat Hospital, Ahvaz University of Medical Sciences, Ahvaz, IR Iran

**Keywords:** Neoplasms, Kidney Transplantation, Cumulative Trauma Disorders, Risk

## Abstract

**Background:**

Malignancy is a common complication after renal transplantation. Death with functioning graft and chronic graft loss are two competing outcomes in patients with post-transplant malignancies.

**Objectives:**

The purpose of our study was to evaluate the risk factors associated with cumulative incidence of these two outcomes.

**Patients and Methods:**

Fine-Gray model was used for 266 cases with post-transplant malignancy in Iran. These patients were followed-up from the diagnosis until the date of last visit, chronic graft loss, or death, subsequently.

**Results:**

At the end of the study, as competing events, chronic graft loss and death with functioning graft were seen in 27 (10.2%) and 53 cases (19.9%), respectively, while 186 cases (69.9%) were accounted as censored. The incidence rate of death was approximately two-time of the incidence rate of chronic graft loss (8.6 vs. 4.4 per 100 person-years). In multivariate analysis, significant risk factors associated with cumulative incidence of death included age (P < 0.007, subhazard ratio (SHR) = 1.03), type of cancer (P < 0.0001), and response to treatment (P < 0.0001, SHR = 0.027). The significant risk factors associated with cumulative incidence of chronic graft loss were gender (P = 0.05, SHR = 0.37), treatment modality (P < 0.0001), and response to treatment (P = 0.048, SHR = 0.47).

**Conclusions:**

Using these factors, nephrologists may predict the occurrence of graft loss or death. If the probability of graft loss was higher, physicians can decrease the immunosuppressive medications dosage to decrease the incidence of graft loss.

## 1. Background

There are 25000 patients with end stage renal disease (ESRD) in Iran of whom 52.7% and 45.5% benefit from hemodialysis and transplantation, respectively (1, 2). Kidney transplantation improves the quality of life and life span of patients with ESRD requiring renal replacement therapy (3-7). However, these patients face two serious risks: graft loss and several complications sometimes leading to death including cardiovascular disease, infections, and malignancies. Immunosuppressive agents have successfully reduced the risk of rejection; however, complications are increasing (8, 9).

One of the common complications after renal transplantation is malignancy. It is the second cause of death in recipients with renal transplantation (6) and it is expected that cancer-associated mortality would become the first cause of death within the next two decades. The overall reported post-transplant malignancy incidence varies from 2% to 31%; however, it happens in a percentage as high as 34% to 50% among renal transplant recipients (RTRs) followed for longer than 20 years (9). In general, the risk of developing malignancy in organ transplants is three to four times greater than general population and the risk of certain types of cancer is as high as 20 to 500 folds (5, 10, 11). Despite the high incidence of skin cancers in RTRs, these tumors are not usually fatal. Solid organ cancers, although less common, are associated with a far worse prognosis in these patients (12).

One year survival of graft after kidney transplantation is 94.7 % in Iran (13). In several studies, death with functioning graft (DWFG) has been reported to occur in 9% to 30% of patients (14-17) and thus, it is accounted for a substantial fraction of graft loss. In most series, consisting mainly of renal transplantations performed in the 1970s to mid-eighties, infection was often reported as the leading cause of death (18-23). Risks and causes of mortality might have changed because of more recent advances in immunosuppressive protocols, improved surgical techniques, and the availability of newer medications for medical treatment of associated risk factors such as hypertension and hyperlipidemia (24).

Nowadays, survival of RTRs is one of the most important concerns. The causes of graft loss have also changed over the time; currently, DWFG and chronic rejection are the principal causes of graft loss (25, 26).

Several pre- and post-transplant markers predict chronic graft loss and death after transplantation. Recipient factors include age, gender, BMI (kg/m^2^), race, cause of renal failure, induction therapy, and use of mycophenolate mofetil, sirolimus and/or calcineurin inhibitors, acute rejection episodes and any treated rejection episode (27), delayed graft function, black race, and recurrence of glomerular disease (28). Donor factors include BMI (kg/m^2^), creatinine (mg/dL), HLA mismatch, age, gender, race, donor-recipient relationship, and type of operation procedure (open vs. laparoscopic) (27). in addition, donor factors affecting long-term post-transplantation graft survival include age, race, sex, cause of death, cold ischemia time, HLA matching, organs from expanded-criteria donors, and cytomegalovirus (CMV) infection (25).

Chronic graft loss and DWFG are the two competing outcomes in RTRs with post-transplant malignancy. Some of RTRs do not progress to chronic graft loss because death precedes it. Hence, preparations recommended before chronic graft loss would be unsuccessful and costly. The factors associated with incidences of these two outcomes in this population are important. When a person in this population experiences death before graft loss, the probability of experiencing graft loss is frequently altered (29).

## 2. Objectives

The purpose of our long-term, prospective, longitudinal study was to evaluate competing risk of chronic graft loss versus DWFG in RTRs with a diagnosed malignancy, and to evaluate the risk factors associated with these two outcomes.

## 3. Patients and Methods

Behzad Einollahi et al. conducted a large multicenter study on 12525 RTRs, accounting for up to 59% of all kidney transplantation in Iran during 22 years follow-up period since October 1984 until December 2008. They collected 266 (2%) biopsy-proven malignancy cases of 26 different types from 16 renal transplant centers in Iran. This study was approved by the local Ethics Committee of Baqiyatallah University of Medical Sciences (30). Our study assessed the incidence of DWFG and chronic graft loss in RTRs with malignancy. The duration of study was 22 years and patients were followed-up from diagnosis of malignancy until death, chronic graft loss, or the date of last visit. Patients with other organ transplants, history of previous malignancy and transplantation from deceased donors with a previous history of malignancy were excluded.

### 3.1. Definition

1. Treatment modalities were considered according to the type of cancer, staging of disease, and involved organs. Management included a combination of reduction, withdrawal or changing of the immunosuppressive agents, chemotherapy, radiotherapy, hormone therapy, and surgical resection.

2. Non Kaposi’s sarcoma tumors (non-KS) included squamous cell carcinoma (SCC), basal cell carcinoma (BCC), and melanoma.

3. Tumors of breast, ovary, and uterine in females, prostate and seminoma in males, and renal cell carcinoma (RCC) and transitional cell carcinoma (TCC) of bladder in both genders were considered as genitourinary and reproductive system (GU and RS) neoplasms.

4. The term of solid tumor was used for all the malignancies except for the skin tumors, post-transplantation lymphoproliferative disorder (PTLD), and GU and RS cancers.

5. Patients with tumor were categorized into five groups according to their type of neoplasm: Non-KS, KS, PTLD, GU and RS tumors, and solid tumors.

6. Monoclonal antibody (ATG/ALG) was required for induction therapy and acute steroid-resistant rejection episodes during the first three months following kidney transplantation. Induction therapy with ATG/ALG was used for highly sensitized patients, those receiving kidneys from deceased donors with delayed graft function, patients with poorly matching living donors, and patients with the second or more transplants. None of the patients took OKT3.

### 3.2. Immunosuppression Protocols

The immunosuppressive therapy was based on cyclosporine/sirolimus, mycophenolate mofetil (MMF)/azathioprine (AZA), and steroids. Before 2000, patients received dual maintenance immunosuppression with prednisone and cyclosporine/AZA or triple therapy with cyclosporine, prednisone, and AZA. Afterwards, most patients received cyclosporine, prednisone, and MMF (31).

### 3.3. Statistical Analysis

In survival models, each studied person could experience one of the several different types of events over the follow up period. Survival times are defined as the time until occurrence of one competing event preventing other event to occur. With competing risks data, the cause-specific hazard measured the instantaneous failure rate due to one risk at a time. It is routinely estimated by constructing the Cox models on cause-specific hazards and treating time to event from the other competing risks as censored with constant hazards (32, 33).

Fine and Gray (31) proposed a regression modeling applied directly on a cumulative incidence function (CIF) for particular use in competing risks analysis which extends the Cox proportional hazards model to competing-risks data by considering the sub-distribution hazard (34). For any event type, this approach focuses on the hazard associated with the CIF, which expresses the effect of covariates directly on the CIF. At time t, the CIF defined the probability of having outcome by time t, while other participants had experienced other events. The CIF for cause k, depends not only on the hazard of cause k, but also on the hazards of all other causes. As opposed to a cause-specific analysis, which would censor the competing event (s), the Fine-Gray approach does not censor them (35, 36). The strength of the association between each predictor variable and the outcome was assessed using the subhazard ratio (SHR), which is the ratio of hazards associated with the cumulative incidence function (CIF) (37). Standard errors of the Fine-Gray model are robust (Huber-White type) and formal check of proportionality by using time-varying covariate effect (34). The Fine-Gray model was implemented in Stata statistical software (V11, 2009; College Station, TX) using the “stcrreg” module.

Chronic graft loss and DWFG are the two competing outcomes in RTRs with post-transplant malignancy over the follow-up period. Survival time (response variable) for any patient was the time from diagnosis of malignancy until either chronic graft loss or death. Therefore, the purpose of our study was to evaluate risk factors associated with both cumulative incidence of death with functioning graft and chronic graft loss by using the Fine and Gray model. The sub-distribution hazard ratios were also determined by Stata 11 software. P < 0.05 was considered as the significant level. Quantitative variables were expressed as mean ± SD, whereas qualitative variables were shown as number and percentage. In addition, we estimated unadjusted incidence rates for progression to DWFG and chronic graft loss as per 100 person-years. It was also described regarding covariates. Included candidates predictors in the model were gender, age, type of cancer, transplantation until diagnosis (month), age at diagnosis, ALG/ATG, treatment modality, response to the treatment, metastasis, CMV infection after cancer, immunosuppressive therapy, and blood group.

## 4. Results

The baseline characteristics of RTRs with malignancy and incidence of competing risk events are displayed in Table 1. The patients with malignancy were followed up after the diagnosis of cancer for a median follow-up period of 22 months (minimum of one month and maximum of 168 months). The male to female ratio was 2.1:1. The mean age of patients was 46.2 ± 12.9 years (range 12-72 years). The mean age at tumor diagnosis was 50.8 ± 13.2 years (range 15.5-82.0 years), and the average time between transplantation and detection of malignancy was 51.08 ± 48.6 months (median 36, range 1-284 months).

**Table 1.** Incidence of Competing Risk Events According to Baseline Characteristics of 266 Post-Transplant Malignancies in Living Kidney Transplant Recipients ^a^, ^b^

Variables	Total	Death Outcome	Chronic Lost Graft Outcome
**Gender**			
Male	180 (67.7)	35 (8.4)	23 (5.5)
Female	86 (32.3)	18 (9.0)	4 (2.0)
**Cancer**			
KS	84 (31.6)	8 (3.5)	10 (4.4)
Non-KS	57 (21.4)	1 (0.5)	1 (0.5)
PTLD	72 (27.1)	23 (20.0)	7 (6.0)
GU & RS	25 (9.4)	8 (20.0)	4 (10.0)
Solid	28 (10.5)	13 (43.0)	5 (16.0)
**ALG/ATG**			
No	183 (83.6)	37 (8.0)	15 (3.0)
Yes	36 (16.4)	10 (15.0)	6 (9.0)
**Treatment modality**			
Without	80 (38.6)	20 (11.0)	22 (12.0)
Decrease	74 (35.7)	11 (6.0)	3 (2.0)
Changed	29 (13.5)	2 (4.0)	0 (0.0)
Unmodified	25 (12.1)	5 (11.0)	1 (2.0)
**Response to treatment**			
No	74 (31.4)	35 (46.0)	11 (14.0)
Yes	162 (68.6)	3 (0. 6)	12 (2.0)
**Metastasis**			
No	156 (75.7)	17 (4.0)	9 (2.0)
Yes	50 (24.3)	21 (25.0)	10 (12.0)
**CMV infection after cancer**			
No	43 (78.2)	7 (5.0)	6 (4.0)
Yes	12 (21.8)	2 (5.0)	2 (5.0)
**Immunosuppressive**			
MMF	96 (38.7)	12 (6.0)	5 (3.0)
AZA	152 (61.3)	41 (11.0)	18 (5.0)
**Blood group**			
O	26 (43.3)	7 (9.0)	1 (1.0)
B	9 (15.0)	1 (2.0)	1 (2.0)
A	16 (26.7)	6 (19.0)	3 (9.0)
AB	9 (15.0)	1 (5.0)	0 (0.0)
**Age, y**	46.2 ± 12.9	46.8 ± 11.8	43.3 ± 11.4
**Transplantation until diagnosis, m**	51.08 ± 48.6	53.8 ± 46.6	48.6 ± 60.2
**Age at diagnosis, y**	50.8 ± 13.2	51.9 ± 11.1	47.6 ± 13.8

^a^ Abbreviation: ALG/ATG, antilymphocyte/antithymocyte globulin; AZA, azathioprine; CMV, cytomegalovirus; GU & RS, genitourinary and reproductive system; KS, Kaposi’s sarcoma; MMF, mycophenolate mofetil; Non-KS, non-Kaposi’s sarcoma; PTLD, post transplantation lymphoproliferative disorder.

^b^ Data are presented in No. (%) and mean ± SD.

### 4.1. Unadjusted Incidence Rate of Competing Risks

Finally, chronic graft loss and DWFG were detected in 27 (10.2%) and 53 cases (19.9%), respectively, and 186 cases (69.9%) accounted as censored. The incidence rate of chronic graft loss was 4.4 per 100 person-years, while the incidence rate of DWFG was 8.6 per 100 person-years. Therefore, the incidence rate of death was approximately two-time the incidence rate of chronic graft loss. Table 1 shows unadjusted incidence of competing risk events of 266 post-transplant malignancies according to baseline characteristics. According to Table 1, incidence of death and chronic graft loss were higher in women and men ordinarily. These incidences are higher in solid cancers, ATG/ALG and AZA treatment regimen, withdrawal of immunosuppressant medications, no response to the treatment, metastasis of tumor, CMV infection after cancer, and blood group type A. The mean age at the time of cancer diagnosis in patients who died was higher than patients with chronic graft loss (51.9 ± 11.1 and 47.6 ± 13.8 years, respectively). The average time from transplantation until the diagnosis of cancer in patients who died was higher than those with chronic graft loss (53.8 ± 46.6 versus 48.6 ± 60.2 months).

### 4.2. Risk Factors for Cumulative Incidence of Death With Functioning Graft and Chronic Graft Loss

Table 2 shows the subhazard ratios (SHR) and standard errors of risk factors estimated by using the univariate Fine and Gray model. Table 3 shows the same results but in a multivariable model. Univariate analyses indicated that the significant risk factors associated with cumulative incidence of death are type of cancer (P < 0.0001), response to the treatment (P < 0.0001, SHR = 0.017), metastasis (P < 0.0001, SHR = 4.42), and immunosuppressive therapy (P = 0.032, SHR = 2.02). Hazard of non-KS cancer was similar to KS cancers, but PTLD, GU and RS, and solid cancers increased the hazard of death compared to KS. In contrary to metastasis of tumor and treatment with AZA, response to the treatment decreased the incidence of death (Table 2). 

The univariate analyses also indicated that the significant risk factors associated with cumulative incidence of chronic graft loss were response to the treatment (P = 0.016, SHR = 0.39), metastasis (P < 0.003, SHR = 3.8), and treatment modality (P = 0.0001). Unlike metastasis of tumor, response to the treatment decreased the incidence of chronic graft loss. The incidence of chronic graft loss in changed and unmodified treatment modalities was similar to the incidence of patients with immunosuppression withdrawal (i.e. without group), while decrease of immunosuppressive drugs decreased the incidence of chronic graft loss compared to withdrawal of immunosuppressant medication. Decreasing incidence of graft loss in women (P = 0.06, SHR = 0.36) and increasing this incidence in patients treated with ALG/ATG (P = 0.07, SHR = 2.34) was approximately significant (Table 2). 

**Table 2.** Factors Associated With Death Versus Chronic Graft Loss as Competing Risks of 266 Post-transplant Malignancies in Living Kidney Transplant Recipients With Univariate Fine and Gray Model ^a^

Variables	Death Outcome			Chronic Lost Graft Outcome		
	SHR	SE	P Value	SHR	SE	P Value
**Gender**	-	-	-	-	-	-
Male	Base Category	-	-	-	-	-
Female	1.1	0.32	0.73	0.36	0.19	0.065
**Age**	1.002	0.01	0.8	0.98	0.01	0.11
**Cancer**	-	-	-	-	-	-
KS	Base Category	-	0.000	-	-	0.13
Non-KS	0.16	0.17	0.088	0.13	0.13	0.048
PTLD	4.28	1.7	0.000	0.91	0.44	0.84
GU & RS	3.78	1.7	0.005	1.53	0.88	0.45
Solid	6.93	3.007	0.000	1.91	1.03	0.23
**Transplantation until diagnosis, m**	1.006	0.009	0.62	0.99	0.006	0.77
**Age at diagnosis**	1.005	0.009	0.55	0.98	0.01	0.14
**ALG/ATG**	-	-	-	-	-	-
No	Base Category	-	-	-	-	-
Yes	1.48	0.52	0.26	2.34	1.12	0.07
**Treatment modality**	-	-	-	-	-	-
Without	Base Category	-	0.21	-	-	0.000
Decrease	0.56	0.21	0.12	0.14	0.08	0.001
Changed	0.27	0.2	0.08	0.14	0.15	0.06
Unmodified	0.88	0.44	0.8	0.16	0.16	0.077
**Response to the treatment**	-	-	-	-	-	-
No	Base Category	-	-	-	-	-
Yes	0.017	0.01	0.000	0.39	0.15	0.016
**Metastasis**	-	-	-	-	-	-
No	Base Category	-	-	-	-	-
Yes	4.42	1.42	0.000	3.8	1.72	0.003
**CMV infection after cancer**	-	-	-		-	-
No	Base Category	-	-	-	-	-
Yes	0.98	0.76	0.97	0.99	0.75	0.99
**Immunosuppressive**	-	-	-	-	-	-
MMF	Base Category	-	-	-	-	-
AZA	2.02	0.67	0.032	2.04	1.03	0.16
**Blood Group**	-	-	-	-	-	-
O	Base Category	-	0.42	-	-	0.55
B	0.38	0.41	0.37	2.5	3.4	0.5
A	1.6	0.87	0.38	5.1	5.9	0.15
AB	0.42	0.47	0.43	3.1	4.47	0.43

^a^ Abbreviations: ALG/ATG, antilymphocyte/antithymocyte globulin; AZA, azathioprine; CMV, cytomegalovirus; GU & RS, genitourinary and reproductive system; KS, Kaposi’s sarcoma; MMF, mycophenolate mofetil; Non-KS, Non Kaposi’s sarcoma; PTLD, post-transplantation lymphoproliferative disorder.

**Figure 1.**
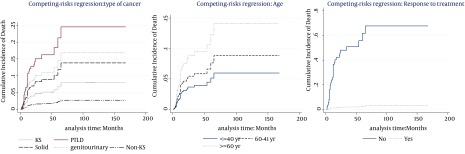
Cumulative Incidence of Death (1A): Type of Cancer (1B), Age (1C), Response to the Treatment

**Figure 2.**
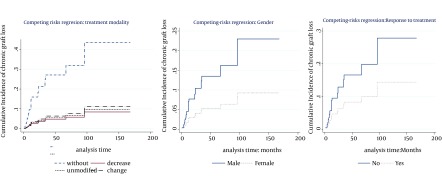
Cumulative Incidence of Chronic Graft Loss (2A), Treatment Modality (2B), Gender (2C): Response to the Treatment

The multivariable model (Table 3) showed that the cumulative incidence of death was related to age (P < 0.007, SHR = 1.03), type of cancer (P < 0.0001), and response to the treatment (P < 0.0001, SHR = 0.027). Hence, at the presence of these factors, no other factor was significant. Table 3 also indicates that the significant risk factors associated with cumulative incidence of chronic graft loss were gender (P = 0.05, SHR = 0.37), treatment modality (P < 0.0001), and response to the treatment (P = 0.048, SHR = 0.47). No other factor presented significant association at the presence of these mentioned factors. Figures 2 and 3 show cumulative incidence of death and cumulative incidence of graft loss for covariates of multivariate model, respectively. According to the figures, hazard of death for Non-KS and solid cancers were similar to KS cancers. Nevertheless, PTLD and GU & RS cancers increased the hazard of death compared to KS cancers (Figure 1A). The incidence of chronic graft loss in changed and unmodified treatment modalities were similar to the incidence of patients with withdrawal of immunosuppression, while decreasing immunosuppressive drugs decreased the incidence of chronic graft loss versus withdrawal of immunosuppressant (Figure 2A). Increasing age, increased the hazard of death (Figure 1B). The incidence of graft loss in women was lower than men (Figure 2B). Response to the treatment decreased the incidence of death and chronic graft loss (Figure 1C and 2C).

**Table 3.** Factors Associated With Death Versus Chronic Graft Loss as Competing Risks of 266 Post-transplant Malignancies in Living Kidney Transplant Recipients With Multivariate Fine and Gray Model^a^

Variables	Death Outcome			Chronic Lost Graft Outcome		
	SHR	SE	P Value	SHR	SE	P Value
**Gender**	-	-	-	-	-	-
Male	-	-	-	Base Category	-	-
Female	-	-	-	0.37	0.2	0.05
**Age**	-	1.03	0.01	0.007	-	-
**Cancer**	-	-	-	-	-	-
KS	Base Category	-	-	-	-	-
Non-KS	0.33	0.33	0.27	-	-	-
PTLD	3.37	1.45	0.005	-	-	-
GU & RS	2.2	0.82	0.03	-	-	-
Solid	1.78	0.75	0.17	-	-	-
**Treatment modality**	-	-	-	-	-	-
Without	-	-	-	Base Category	-	-
Decrease	-	-	-	0.15	0.09	0.002
Changed	-	-	-	0.21	0.22	0.14
Unmodified	-	-	-	0.18	0.19	0.1
**Response to the treatment**	-	-	-	-	-	-
No	Base Category	-	-	-	-	-
Yes	0.027	0.017	0.000	0.47	0.18	0.048

^a^ Abbreviation: ALG/ATG, antilymphocyte/antithymocyte globulin; AZA, azathioprine; CMV, cytomegalovirus; F, Fisher; GU & RS, genitourinary and reproductive system; KS, Kaposi’s sarcoma; MMF, mycophenolate mofetil; Non-KS, Non Kaposi’s sarcoma; PTLD, post-transplantation lymphoproliferative disorder

## 5. Discussion

The aim of our study was to identify the risk factors leading to death or graft loss in RTRs in whom malignancy was developed. Therefore, the risk factors associated with other complications were omitted. Prior studies did not considered cancer cases in a model of competing risks and might not provide the necessary decision-making tools for practicing nephrologists on this population. Most estimates of mortality versus chronic graft loss were derived from all recipients. Whereas these estimates are undoubtedly important in the context of management of renal recipients, they offer no guidance to the practicing nephrologists to manage cancer cases of recipients. Establishing risk factors accelerating chronic graft loss and distinguishing them from those that increase mortality would be an important research goal. If the risk factors are different for these competing end-points, then the practice of graft loss with mortality as a composite outcome may be questionable since they might share different pathways.

For this study, we used data of multicenter study that was conducted by Einollahi et al. They concluded that the skin cancer (52.9%) was the most frequently observed malignancy after renal transplantation including KS, SCC, BCC, and melanoma, followed by PTLD (27%); whereas GU & RS tumors (9.4%) were the most common malignancy among the other visceral tumors. In addition, Einollahi et al. showed that the best survival was observed in SCC and BCC, and the worst was seen in non-hematologic and non-skin tumors. PTLD was the most common cause of death in both genders and in all age groups. In this study, the incidence of cancer in men was greater than women, and the most frequent tumor in men and women was KS. Induction therapy with ATG/ALG was used only for 16.4% of patients. AZA-based regimens was used in approximately 61.3% of patients (152 cases), while the rest were on MMF-based therapy (96 patients; 38.7%). Regarding the treatment modalities, 162 (68.6%) cases had responded to the treatment. Most patients received a kidney from a living unrelated donor (87.5%), followed by 9.8% living related and 2.7% deceased donor transplantation (30). However, the major findings of our analysis are as follows:

1. The incidence rate of death was approximately two times the incidence rate of chronic graft loss (8.6 versus 4.4 per 100 person-years).

2. The incidence of death was higher in older patients, PTLD, and GU & RS tumor cases and hazard of death for Non-KS and solid cancers were similar to KS cancer. Response to the treatment decreased the incidence of death. Our estimates extended the findings of Mazuecos et al. and Einollahi et al. who reported that age and immunosuppressive treatment were related to cancer development (26, 30, 38) that increased mortality. Moreover, Alonso and Oliver showed in their study that post-transplant mortality was dependent on age in all recipients (39). We focused in our study on recipients with cancer. In addition, we showed in our univariate analysis that metastasis and treatment with AZA increased the cumulative incidence of post-transplant death.

3. The incidences of chronic graft loss was higher in males and was lower in lower dosage of immunosuppressive drugs. It remained similar by regimen change, modification, or withdrawal of immunosuppressant. Response to the treatment decreased the incidence of this outcome. In addition, we showed in our univariate analysis that metastasis increased the cumulative incidence of chronic graft loss. Tiong et al. and Harada et al. showed in their studies that acute rejection episodes and any treated rejection episode (27), delayed graft function, black race, and recurrence of glomerular disease were independent risk factors of graft loss (28). Moreover, donor risk factors affecting long-term post-transplantation graft survival included age, small donor size with large recipient size, race, sex, cause of death, cold ischemia time, female donor gender, HLA mismatch, organs from expanded-criteria donors, and cytomegalovirus (CMV) status (25, 27). Briganti et al. showed that predictive factors of decreased 12-month graft survival on univariate analysis were older recipient age, presence of vascular disease in the recipient at the time of initiation of renal replacement therapy, higher peak panel reactive antibody levels, longer time on dialysis prior to transplantation, older donor age, cadaveric donor source, brain damage as the cause of donor death, greater number of human lymphocyte antigen (HLA) mismatch, longer cold ischemic time, and earlier year of transplantation (40).

### 5.1. Limitation

Einollahi's study data was collected from previous medical records; thus, we had some missing data. In addition, some cases were not followed up until reaching death or graft loss and our censored data was 69.9%. Therefore, for increasing the power of this model, it is suggested to use studies with less missing and censored data.

Using estimates provided in this study can guide us in the following ways. Patients who are older, have PTLD, GU, or RS tumor, or did not respond to the treatment are more likely to die, while patients who are female, with decreased immunosuppressant regimen, or have response to the treatment are less likely to reach chronic graft loss.

The response to the treatment is a decreasing factor for the incidence of both endpoints, and does not distinguish between them. Nevertheless, factors such as age, gender, treatment modality, and type of cancer might discriminate between them. Therefore, by using these factors, nephrologists might be able to predict the occurrence of graft loss or death and if the probability of graft loss was higher, they would decrease the immunosuppressive drug dosage to decrease the incidence of graft loss.
